# Supplementary material for: Modeling glioblastoma heterogeneity as a dynamic network of cell states
Source: Mol Syst Biol. 2021 Sep 16;17(9):e10105. doi: 10.15252/msb.202010105 (PMC8444284; doi:10.15252/msb.202010105)
Supplement: Supplementary file 6 — Source Data for Figure 5 [file MSB-17-e10105-s004.zip › Figure5A_sourcedata/GSEA_3071/nosigdb_stateVI.GseaPreranked.1622795169056/DALMO_EXTINCTION_UNPUB.html]

Details for gene set DALMO\_EXTINCTION\_UNPUB[GSEA]

|  || Dataset | state63071 |
| Phenotype | NoPhenotypeAvailable |
| Upregulated in class | na\_pos |
| GeneSet | DALMO\_EXTINCTION\_UNPUB |
| Enrichment Score (ES) | 0.48975304 |
| Normalized Enrichment Score (NES) | 2.1587865 |
| Nominal p-value | 0.00591716 |
| FDR q-value | 0.0025705593 |
| FWER p-Value | 0.015 |
Table: GSEA Results Summary

  

Fig 1: Enrichment plot: DALMO\_EXTINCTION\_UNPUB      
 Profile of the Running ES Score & Positions of GeneSet Members on the Rank Ordered List

  

| PROBE | GENE SYMBOL | GENE\_TITLE | RANK IN GENE LIST | RANK METRIC SCORE | RUNNING ES | CORE ENRICHMENT || 1 | THBS1 |  |  | 12 | 1.097 | 0.0804 | Yes |
| 2 | CAV1 |  |  | 13 | 1.087 | 0.1635 | Yes |
| 3 | LOX |  |  | 29 | 0.968 | 0.2332 | Yes |
| 4 | PLAUR |  |  | 34 | 0.936 | 0.3036 | Yes |
| 5 | GLIPR1 |  |  | 81 | 0.781 | 0.3505 | Yes |
| 6 | PRSS23 |  |  | 108 | 0.732 | 0.3992 | Yes |
| 7 | BACE2 |  |  | 249 | 0.563 | 0.4035 | Yes |
| 8 | CNN2 |  |  | 349 | 0.512 | 0.4153 | Yes |
| 9 | GPRC5A |  |  | 371 | 0.502 | 0.4478 | Yes |
| 10 | CTSB |  |  | 471 | 0.464 | 0.4559 | Yes |
| 11 | BNC2 |  |  | 477 | 0.462 | 0.4898 | Yes |
| 12 | IL7R |  |  | 718 | 0.392 | 0.4534 | No |
| 13 | ARPC1B |  |  | 847 | 0.369 | 0.4462 | No |
| 14 | CTSC |  |  | 1767 | 0.254 | 0.2117 | No |
| 15 | COL15A1 |  |  | 2379 | -0.349 | 0.0696 | No |
| 16 | TGFBI |  |  | 2679 | -0.419 | 0.0190 | No |
| 17 | LUM |  |  | 2775 | -0.448 | 0.0270 | No |
| 18 | COL3A1 |  |  | 3430 | -0.960 | -0.0804 | No |
| 19 | COL1A2 |  |  | 3602 | -1.803 | 0.0099 | No |
Table: GSEA details [plain text format]

  

Fig 2: DALMO\_EXTINCTION\_UNPUB: Random ES distribution      
 Gene set null distribution of ES for **DALMO\_EXTINCTION\_UNPUB**
